# Hemodynamic assessment in children after cardiac surgery: A pilot study on the value of infrared thermography

**DOI:** 10.3389/fped.2023.1083962

**Published:** 2023-04-05

**Authors:** Armelle Bridier, Monisha Shcherbakova, Atsushi Kawaguchi, Nancy Poirier, Carla Said, Rita Noumeir, Philippe Jouvet

**Affiliations:** ^1^ Pediatric Intensive Care Unit, CHU Sainte-Justine, University of Montreal, Montreal, QC, Canada; ^2^ Department of Electrical Engineering, École de Technologie Supérieure of Montreal, Montreal, QC, Canada; ^3^ Department of Intensive Care Medicine, Tokyo Women's Medical University, Tokyo, Japan; ^4^ CHU Sainte-Justine Research Center, Montréal, QC, Canada

**Keywords:** infrared thermography, cardiac surgery, pediatric, low cardiac output state, hemodynamic monitoring

## Abstract

**Introduction:**

Low cardiac output syndrome in the postoperative period after cardiac surgery leads to an increase in tissue oxygen extraction, assessed by the oxygen extraction ratio. Measurement of the oxygen extraction ratio requires blood gases to be taken. However, the temperature of the skin and various parts of the body is a direct result of blood flow distribution and can be monitored using infrared thermography. Thus, we conducted a prospective clinical study to evaluate the correlation between the thermal gradient obtained by infrared thermography and the oxygen extraction ratio in children at risk for low cardiac output after cardiac surgery.

**Methods:**

Children aged 0 to 18 years, having undergone cardiac surgery with cardio-pulmonary bypass in a pediatric intensive care unit were included in the study. One to 4 thermal photos were taken per patient using the FLIR One Pro thermal imaging camera. The thermal gradient between the central temperature of the inner canthus of the eye and the peripheral temperature was compared to the concomitant oxygen extraction ratio calculated from blood gases.

**Results:**

41 patients were included with a median age of 6 months (IQR 3–48) with median Risk Adjustment for Congenital Heart Surgery-1 score was 2 (IQR 2–3). Eighty nine thermal photos were analyzed. The median thermal gradient was 2.5 °C (IQR 1,01–4.04). The median oxygen extraction ratio was 35% (IQR 26–42). Nine patients had an oxygen extraction ratio ≥ 50%. A significant but weak correlation was found between the thermal gradient and the oxygen extraction ratio (Spearman's test *p* = 0.25, *p* = 0.016). Thermal gradient was not correlated with any other clinical or biologic markers of low cardiac output. Only young age was an independent factor associated with an increase in the thermal gradient.

**Conclusion:**

In this pilot study, which included mainly children without severe cardiac output decrease, a significant but weak correlation between thermal gradient by infrared thermography and oxygen extraction ratio after pediatric cardiac surgery was observed. Infrared thermography is a promising non-invasive technology that could be included in multimodal monitoring of postoperative cardiac surgery patients. However, a clinical trial including more severe children is needed.

## 1. Introduction

Low cardiac output syndrome (LCOS) occurs in the post-operative period after cardiac surgery and can affect 25% of patients (1, 2). LCOS results in an insufficient supply of oxygenated blood to the tissues, which leads to an increase in tissue oxygen extraction assessed by the oxygen extraction ratio (O_2_ER). This hemodynamic impairment is associated with an increase in mortality, in duration of mechanical ventilation and PICU length of stay (1, 3). LCOS diagnosis is defined as fall of the cardiac index below 2 L/min per m^2^, but invasive measurement of cardiac index is not usually performed in pediatric cardiac intensive care units (PCICU). Thus, the clinical diagnosis of LCOS mostly relies upon caregivers' qualitative assessment including skin warmth of the extremities and capillary refill time (1, 4).To improve LCOS diagnosis in PCICU, a clinical-biological LCOS score has recently been developed and was correlated with composite measures of postoperative morbidity (5). This score includes the qualitative assessment of recoloration time and the quantitative assessment of toe temperature. It was designed to predict adverse events potentially related to low cardiac output but has not been compared with an objective measure of cardiac output. Instead of toe temperature, several authors have proposed to use a thermal gradient between core and peripheral temperature with a threshold value of 5°C being correlated with decreased cardiac output and increased peripheral vascular resistance (6–8).

Skin warmth can be assessed qualitatively by caregiver touch or quantitatively *via* a skin thermometer or infrared thermography (IRT). IRT is a non-invasive, non-contact thermal measurement tool. The quantification of a thermal gradient by IRT should be more objective and reproducible than clinical assessment. The thermal resolution rises up to 0.02°C. This technology creates an image from the temperature obtained after the conversion of the infrared radiation emanating from a body or an object. IRT is used in many medical conditions (9–12) including assessment of local microcirculation in the extremities in the context of hemodynamic instability (13).

The use of the thermal gradient assessed by IRT to detect a decrease in cardiac output has not been extensively studied in PCICU. The aim of our pilot study was to assess the correlation between thermal gradient obtained by IRT and O_2_ER in children aged 0 to 18 years, following cardiac surgery with cardiopulmonary bypass. We hypothesized that an increase in the thermal gradient obtained by IRT would be positively correlated with the increase in O_2_ER after cardiac surgery, and therefore could be used for early detection of low cardiac output syndrome.

## 2. Material and methods

### 2.1. Study setting

We conducted a prospective observational study in the pediatric intensive care unit (PICU) of Sainte-Justine Hospital, Montreal, Quebec, a quaternary referral center for congenital cardiac disease. In our center,150 to 200 cardiac surgeries are performed annually.

The Sainte-Justine Research Ethics Board (REB) approved the protocol (reference 2021–2854). The eligible patients were identified through screening of the surgical schedule. Written informed consent for video and photo recordings were obtained from the parents or legal tutor before surgery or during the first hour after surgery (MEDEVAC project approved by the REB, reference 2016–1242). The study aimed to evaluate the feasibility of infrared thermography after cardiac surgery. The timing of the thermal picture recording was determined according to the biological analysis timing, allowing the O_2_ER determination. These blood samples were taken according to the patient's clinical needs and hemodynamic stability.

### 2.2. Study population

From January 2020 to March 2021, all children aged 0 to 18 years scheduled for cardiac surgery with cardiopulmonary bypass (CPB) were deemed eligible. Exclusion criteria were the conditions that could modify patients' core temperature: patients on extracorporeal membrane oxygenation support, continuous hemofiltration, and peritoneal dialysis. Other exclusion criteria were conditions that could distort the thermal gradient calculation obtained by IRT: any extensive skin disease, external active heating (frequent in neonates) or cooling system. It should be noted that although neonates had cardiopathies at risk for low cardiac output after surgery, we had to exclude most of them because the heated incubators would have distorted the interpretation of thermal images.

### 2.3. Index test

The thermal gradient was defined as the difference between the temperature of the internal eye canthus and the peripheral temperature taken at the hallux by image-thermography.

Once the patient was admitted to PICU after cardiac surgery, the thermal images were taken within 30 min before or after blood gas sampling. The thermal photos were taken by the clinical fellow on duty after a short training session on how to use the camera. During the 18 h following cardiac surgery, 1 to 4 photos were taken depending on the availability of the clinical fellow. The thermal images were taken with the FLIR-One Pro camera (Teledyne FLIR). The characteristics of this camera were a thermal sensitivity of 0.07°C, a thermal resolution of 19,200 pixels, a temperature range for elements from −20°C to +400°C, and a focal length (focus) of 15 cm or more. We used a standardized protocol as recommended by the American Academy of Thermology.

The peripheral temperature image was obtained of the hallux, and the temperature image equivalent to the central temperature was obtained at the internal canthus of the eye (14) on a naked patient with the extremities and face visible. Pictures that did not fulfill the environmental conditions and the reliability criteria of a thermal image for a medical study were excluded (11). The documented environmental conditions included the room temperature, room humidity and the distance between the camera and the patient. The room temperature was maintained between 21 and 23°C. Humidity was centrally regulated for the whole unit between 30% and 60%. The camera was fixed at 80 to 100 cm from the patient and at a 45° angle to the bed plane. For a given patient, the camera was left at the same place throughout the study to ensure image reproducibility. The maximum temperature captured on the scene had to be higher than 35°C so that the image would faithfully reflect human temperatures. The pictures acquired were stored on the phone connected to the camera, then transferred to a PC and processed by MATLAB software. A temperature scale on each thermal image allowed linkage of a colored pixel to a given temperature. We could use a gray or colored scale. On a gray scale, the black pixel corresponded to 0°C, and the white color corresponded to 255°C. We superimposed the thermal image on the simple digital image when necessary to better determine the regions of interest, i.e., the internal canthus of the eye and the hallux.

### 2.4. Primary outcome: O_2_ER

Our primary outcome was O_2_ER. O_2_ER is a good marker of cardiac output. An increase in O_2_ER corresponds to a mismatch between oxygen demand and delivery (15). Changes in O_2_ER occur earlier than lactate changes during hemodynamic instability. This marker of tissue oxygen extraction is not influenced by heart disease category (cyanogenic or non-cyanogenic). This marker is also more accurate than cardiac output assessed by transthoracic ultrasound because it is not affected by technical issues in the post-operative period and interpersonal variability of ultrasound measurements.

O_2_ER is calculated as (SaO_2_-SvO_2_)/ SaO_2,_ where SaO_2_ is the arterial blood gas oxygen saturation and SvO_2_ is the venous blood gas oxygen saturation collected *via* a central venous catheter in the superior vena cava. For each patient, correct position of the central venous line was checked on the chest x-Ray at PICU admission. For patients with Glenn physiology, the central venous catheter was in the superior vena cava not fully reflecting the venous blood mixture. Normal O_2_ER is 25% to 30% in the superior vena cava. Blood gas samples to calculate O_2_ER were scheduled for each patient according to department protocol and patient stability. There was no additional analysis performed for the study. We divided patients into two groups according to O_2_ER level: “normal and low O_2_ER” group (O_2_ER < 30%) and “high O_2_ER” group (O_2_ER ≥ 30%).

### 2.5. Secondary outcomes

The secondary outcomes were blood lactate level, base excess level (from a blood gas sampled within 30 min before or after the thermal picture was taken, and physiologic parameters including heart rate, cerebral rSO_2_, hourly urine output. Those parameters were documented during routine clinical care and obtained subsequently from the electronic health care record (16).

### 2.6. Other parameters of interest

To describe the population, baseline demographic information (age, weight at the time of surgery, gender), cardiac diagnosis, Risk Adjustment for Congenital Heart Surgery 1 (RACHS-1 score) (17) were obtained from the electronic medical record. Intraoperative data [surgical procedure, cardio-pulmonary bypass (CPB) duration, aortic cross-clamp duration] and transesophageal or transthoracic echocardiography results were obtained from the anesthesia and operating room records. Use of vasoactive drugs was documented. Vasoconstrictor drugs were defined as epinephrine at a dose greater than 0.1 mcg/kg/min (lower doses are routinely used mostly for the inotropic effect) or norepinephrine and phenylephrine at any dose. Vasodilator drug was defined as milrinone at a rate greater than 0.7 mcg/kg/min (lower doses are routinely used mostly for the inotropic effect) or nicardipine at any dose.

### 2.7. Sample size calculation

We calculated a sample size of 40 patients with 120 measurements of O_2_ER and thermal gradient would be required in order to attain a moderate correlation between O_2_ER and thermal gradient with a power of 80% at the 5% alpha threshold.

### 2.8. Statistical analysis

Descriptive statistics were reported as proportion and median (with interquartile range). To examine the correlation between the thermal gradient and O_2_ER, as well as with secondary outcomes (urinary output, lactate level, base excess value, and cerebral rSO_2_), Spearman's test was used (*p*). To assess factors associated with thermal gradient, linear regression analyses were performed. The Wilcoxon-Mann-Whitney test was used to compare the medians of the categorical variables. Variables included in the linear regression were those deemed clinically relevant, including age, the use of vasodilator or vasoconstrictor drugs, the core temperature, and the CPB duration. Any factor in the univariate analysis with a *p* < 0.1 was included in the multivariate analysis. Level of significance was set as *p* < 0.05.

## 3. Results

**Figure 1:**
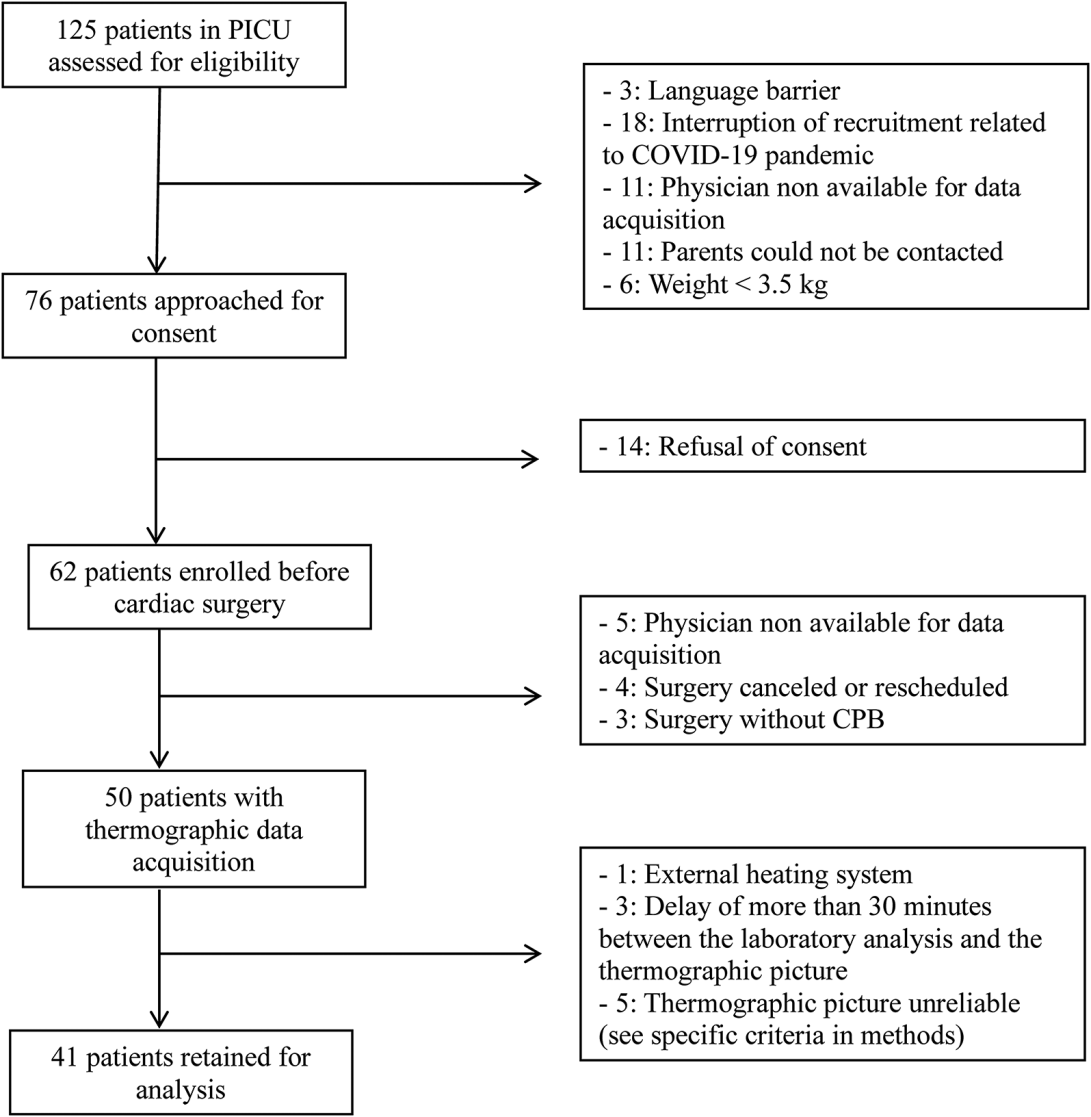
Flow chart.

One hundred twenty-five patients were screened, 50 patients were included and had thermographic data acquisition. A total of 41patients were included in the analysis (Figure 1).

The demographic and baseline clinical characteristics are summarized in Table 1. The median age was 6 months (IQR = 3–47). There was no significant difference for sex. Cardiac malformations were mainly right ventricular outflow tract anomalies (*n* = 12), ventricular septal defect (*n* = 11) (see Table 1). The median PICU length of stay in our cohort was 5 days (IQR = 4–7), with a median duration of mechanical ventilation (MV) of 7 h (IQR = 4–23). Two patients developed a complication potentially related to LCOS such as necrosis enterocolitis. One patient required prolonged MV (2 832 h).

**Table 1:** Patient demographics and baseline characteristics by O_2_ER value.

Characteristics	All patients (*n = *41)	O_2_ER < 30% (*n = *16)	O_2_ER ≥ 30% (*n = *25)	*P* value^a^
Median age (IQR), mo^b^	6 (3–47)	15 (5–96)	5 (2–26)	0,038
Female sex *n* (%)	23 (56)	9 (56)	14 (58)	NS
Risk Adjustment for Congenital Heart Surgery-1^b^, Category, *n* (%)	2 (2–3)	2 (2–3)	2 (2–3)	NS
1	1 (2)	1 (7)	0 (0)	
2	21 (54)	8 (53)	13 (52)	
3	14 (26)	5 (33)	9 (36)	
4	3 (8)	1 (7)	2 (8)	
5	0 (0)	0 (0)	0 (0)	
6	0 (0)	0 (0)	0 (0)	
**Type of repair, *n* (%)**				
Univentricular	2 (5)	1 (6)	1 (4)	NS
Fontan surgery	2			
Biventricular	39 (95)	15 (94)	24 (96)	
Tetralogy of Fallot repair	12			
Ventricular septal defect repair	11			
Interrupted arch repair	4			
Aortic valvuloplasty	3			
CAV repair	2			
APVR repair	2			
Heart transplantation	2			
Pulmonary stenosis	1			
ALCAPA repair	1			
Arterial switch operation	1			
Total CPB time, min^b^	101 (77–125)	104 (81–120)	99 (76–125)	NS
Aortic cross clamp time^b^	69 (48–82)	73 (53–90)	69 (47–83)	NS
Hypothermia during CPB *n* (%)	7 (17)	2 (12)	5 (20)	NS
Decreased Cardiac Function on TEE, *n* (%)	6 (16)	3 (19)	3 (12)	NS
Vasopressor agent, *n* (%)	4 (10)	3 (19)	1 (4)	NS
Vasodilator agent, *n* (%)	11 (26)	5 (30)	6 (24)	NS

CAV: Complete atrioventricular canal, APVR: anomalous pulmonary venous return, ALCAPA: abnormal left coronary artery from pulmonary artery, IQR: interquartile range, CBP: cardiopulmonary bypass, TEE: Transesophageal echocardiography.

^a^ Comparison between patients with O_2_ER ≥ 30% and O_2_ER < 30%.

^b^ Median and interquartile range.

Four patients had vasopressors and 11 patients a vasodilator drug. The capillary refill time evaluated by the nurses was in 100% of the cases lower than 2 s.

The average lactate level was 2.1 mmol/L (±1.9) and the average cerebral rSO_2_ was 68% (±12).

### 3.1. Thermal pictures interpretation

**Figure 2:**
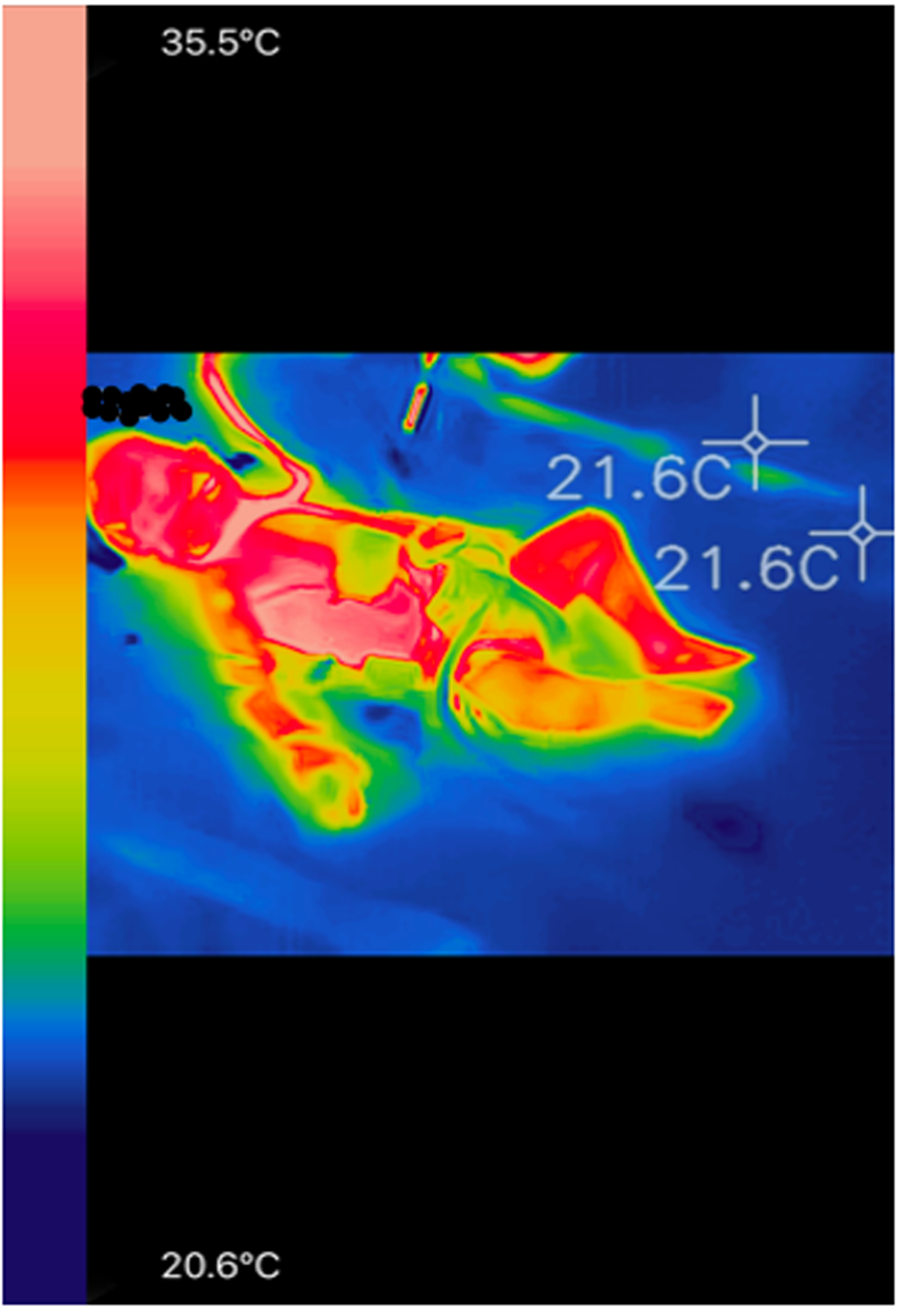
Example of a colored infrared image in a child undergoing postoperative cardiac surgery with the FLIR One Pro camera. Blue pixels correspond to the coldest temperatures and the pale pink pixels correspond to the warmest temperatures, as it is the case in front of the internal cantus of the eye and the thoracic region.

Eighty-nine thermal images were retained for analysis that corresponded to one to four images per patient (image example in Figure 2). The median thermal gradient was 2.49°C (IQR = 1.02–4.04). Forty six percent of the expected thermal images were not taken or did not fulfill the reliability criteria of a thermal image and were excluded from the statistical analysis. Issues included: presence of a heat source around the patient [heated incubator, non-invasive ventilation (NIV) mask], the obstruction or absence of the zones of interest in the picture (obstruction of the face by a NIV mask), maximum temperature below 35°C.

### 3.2. Correlation between the thermal gradient and the O_2_ER

The median O_2_ER was 35% (IQR 26–42). Only 8 patients had an O_2_ER above 50%. Patients with an O_2_ER > 30% were significantly younger than those with an O_2_ER < 30%.

**Figure 3:**
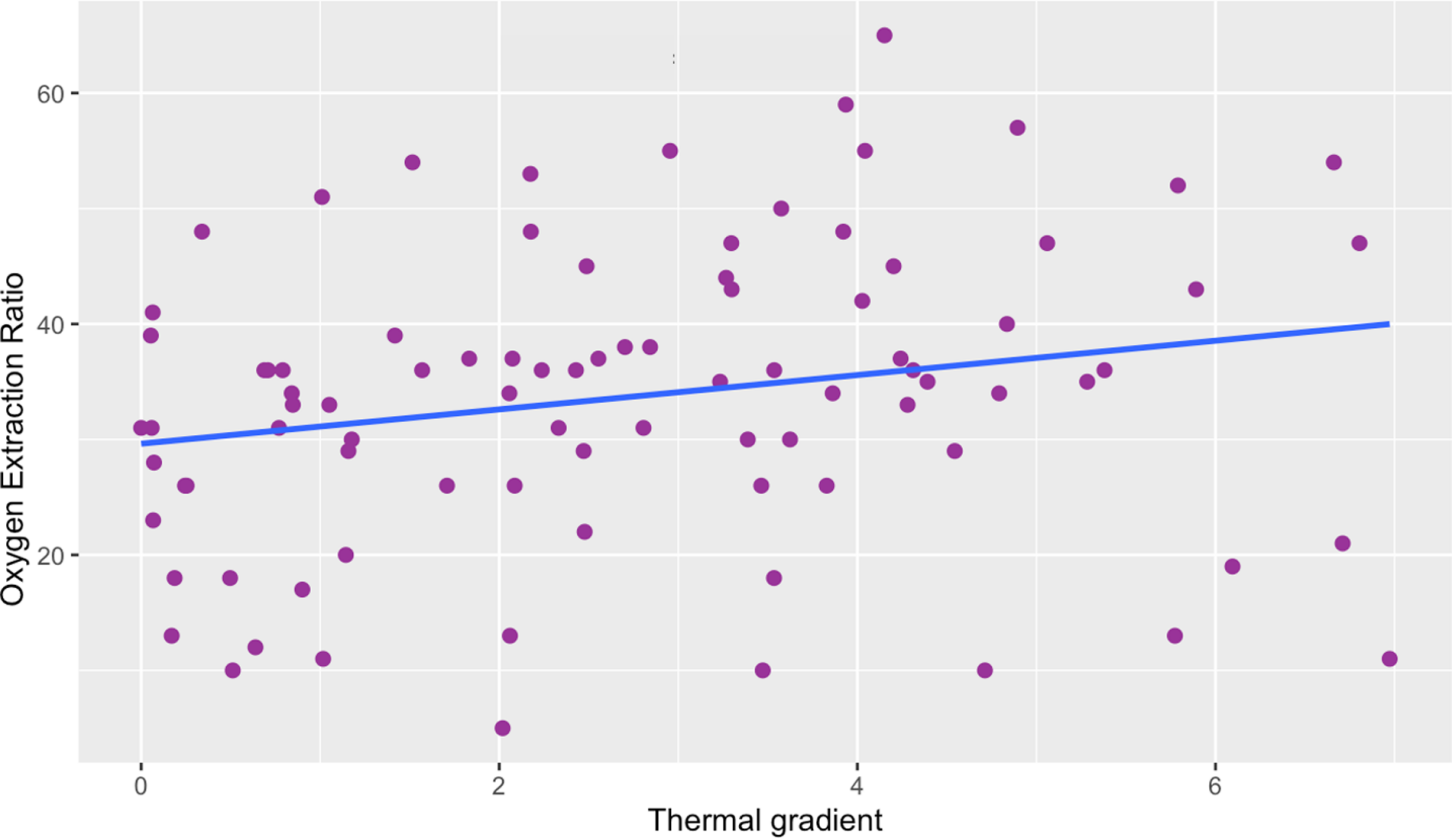
Scatter plot of thermal gradient vs. oxygen extraction ratio. There is a weak correlation between the thermal gradient and the O_2_ER. Thermal gradient is expressed in °C, Oxygen Extraction Ratio is expressed in %. Spearman correlation coefficient was *p *= 0.25, *p *= 0.016.

We found a weak positive correlation between the thermal gradient obtained by IRT and the O_2_ER. The Spearman's correlation coefficient was *p* = 0.25 *p *= 0.016 (Figure 3). Since we had between 1 and 4 data per patient, we studied the correlation using the medians of the data for each patient to take into account possible interindividual variability. We found that the correlation coefficient was not significantly different from that obtained (*p* = 0.21 (*n* = 41) vs. *p* = 0.25 (*n* = 89)).

### 3.3. Correlation between the thermal gradient and the other clinical and biological variables

Among the clinical and biological variables tested, we did not find a significant correlation between the thermal gradient and the following variables: lactate level (*p* = 0.04 *p *= 0.70), base excess (*p* = 0.01 *p *= 0.89), urinary output (*p* = 0.03 *p *= 0.76). There was, however, a significant negative correlation between the cerebral rSO_2_ and the thermal gradient with a Spearman coefficient *p* = − 0.28 *p *= 0.001.

**Figure 4:**
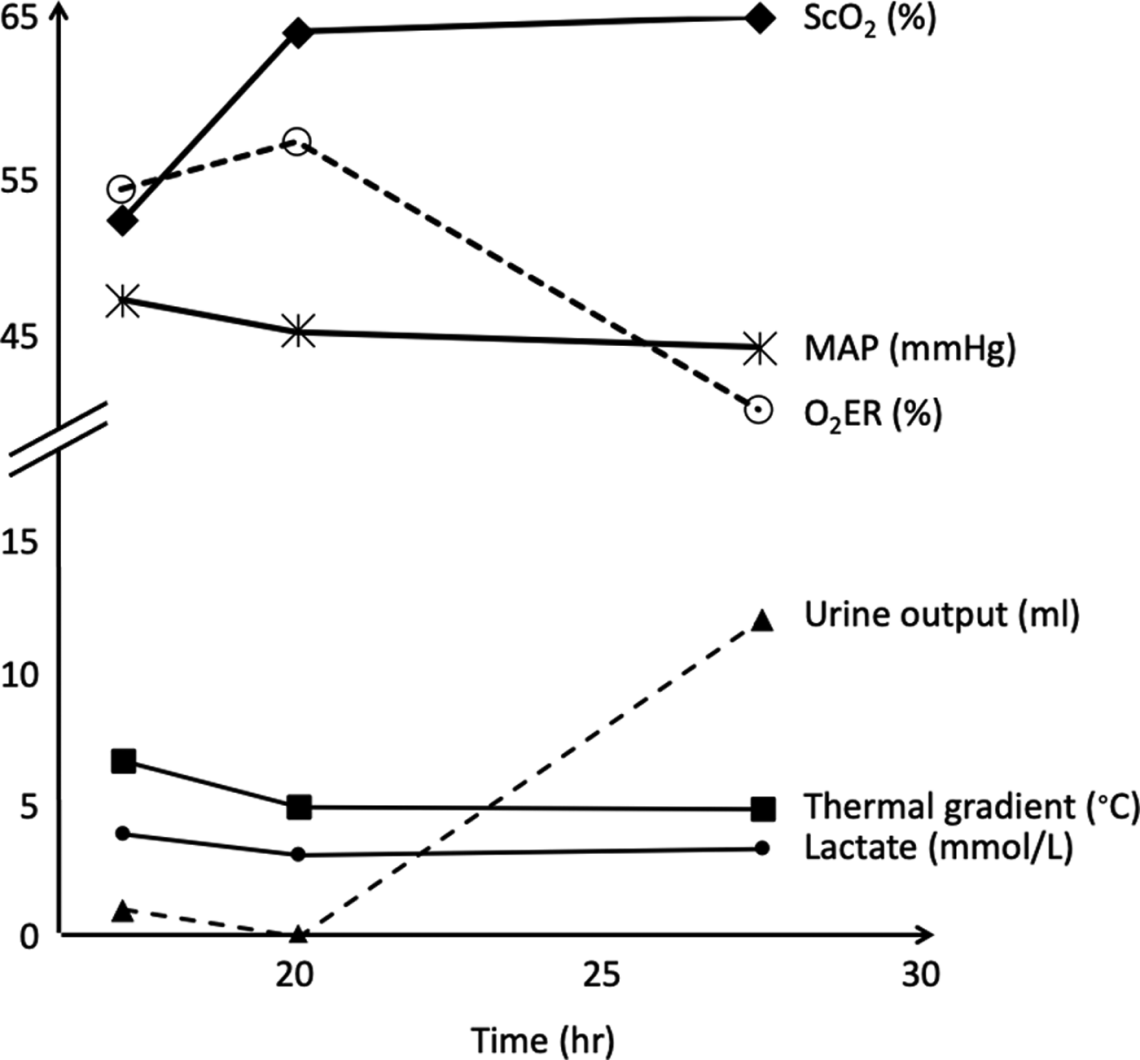
Post- operative follow-up of the thermal gradient and other hemodynamic variables over time for a neonate with a pulmonary atresia and a ventricular septal defect. Thermal gradient showed a parallel evolution to that of the cerebral blood oxygen saturation and blood lactate levels. ScO_2_, cerebral blood oxygen saturation measured by near infrared spectroscopy; MAP, mean arterial pressure; O_2_ER, oxygen extraction ratio.

Follow-up of the thermal gradient over time for a given postoperative cardiac surgery patient showed a parallel evolution to that of the cerebral blood oxygen saturation and blood lactate levels (Figure 4).

### 3.4. Assessment of factors associated with thermal gradient

In the univariate analysis, the only factor that was statistically significant was patient age (*p *= 0.0001). The younger the patient, the greater the thermal gradient. No other factor, among core temperature, CPB duration, vasopressor use or vasodilator use, reached a significance level of *p* < 0.1, so no multivariate analysis was performed.

## 4. Discussion

In the post-operative period following cardiac surgery, we collected thermal gradient measurements in 41 of 50 included patients. Despite a standardized protocol to acquire IRT, we identified several clinical situations that interfere with thermal gradient between the internal canthus of the eye and the hallux measurement including heated incubators and non-invasive ventilation. We found a weak correlation between the thermal gradient obtained by IRT and the O_2_ER. This correlation was higher for patients with O_2_ER values above 30%. The thermal gradient was not correlated with any clinical and biological variables that can be altered in the context of LCOS.

The use of thermal gradient as a tool for monitoring cardiac output is controversial. Older studies found an increase in thermal gradient correlated with an increase in systemic vascular resistance, a decrease in cardiac output, stroke index, and urine output (7, 18, 19). An increase in gradient preceded or was concomitant with changes in hemodynamic parameters (18). Such a correlation has also been found in the adult population (6). Conversely, several other authors did not find any clinically significant correlation (20–23). In most of these studies, thermodilution was used to measure cardiac output, more rarely doppler ultrasound was used (22). However, thermodilution is an invasive technique and is no longer routinely used to assess cardiac output in PICU. O_2_ER is a well described marker of cardiac output (15).We found a weak correlation between O_2_ER and the thermal gradient. Thus, as found in several studies, the thermal gradient correlated poorly with markers of cardiac output. In our study, the median O_2_ER was 35%, only 11 O_2_ER values were above 50% and 1 above 60%. Thus only a few patients had significant evidence of low cardiac output. For the group of highest O_2_ER values, the correlation with thermal gradient increased from weak to medium, suggesting that the thermal gradient becomes more significant as the mechanisms for compensating decreased cardiac output begin to be overwhelmed and when more pronounced peripheral vasoconstriction occurs.

The thermal gradient measurement technique has not always been well described in the literature (6). Only in one study was the thermal measurement performed by infrared thermography. Our pilot study highlights the technical difficulties of using the infrared camera in an intensive care unit, especially the use of incubators and the numerous medical devices used on the face and hallux. Due to the discontinuous nature of blood gas sampling, we chose to perform discontinuous measurements of the thermal gradient. Those technical difficulties resulted in the exclusion of many photos (46%). Quality and consistency of the thermography imaging could be improved by the exclusion of patients with non-invasive ventilation, positioning the camera on the ceiling above the bed (to standardize patient distance and avoid interactions with nursing care), improvement of the spatial resolution of the camera and the automation of thermal picture capture.

There was no correlation between the thermal gradient and serum lactate, base excess, and urine output. Unlike the thermal gradient, these are all late markers of tissue hypoperfusion in the context of LCOS (15). Some authors suggest that global hemodynamic parameters may not be sensitive enough to reflect changes in peripheral blood flow in critically ill patients (24).

However, NIRS allows a real time evaluation of oxygenation and regional vascular perfusion, and a real time estimation of central venous saturation and cardiac output (25–27). This may explain why we found a moderate correlation between cerebral rSO_2_ and the thermal gradient. In one patient with several assessments of the gradient over time, we observed a parallel evolution of the thermal gradient and cerebral rSO_2_, suggesting that the follow-up of the thermal gradient could also give an information on the evolution of the hemodynamic pattern (Figure 4).

Younger patients had a higher thermal gradient. This may be due to greater severity of the cardiac lesions in those operated in the neonatal period. Indeed, neonatal cardiac surgeries often have a higher RACH-1 score and are therefore more at risk of postoperative hemodynamic instability. However, as most of the neonates were placed in a heated incubator, the number of neonates included in this study is too small to allow further analysis of the interaction between thermal gradient and younger age.

Ryan et al. found a statistically significant correlation between the thermal gradient (core temperature assessed by a pulmonary artery thermistor and the foot temperature assessed by thermography) and cardiac output assessed by thermodilution (21).They hypothesized that modifications in systemic vascular resistance with use of vasoactive drugs, for example, may impact this correlation. In our study, we did not find a significant relationship between vasoactive drugs used and the thermal gradient. Neither milrinone, known for its vasodilatory effects, or, on the contrary, vasoconstrictor agents, had a significant impact on the thermal gradient. However, a few patients had high doses of vasoactive agents and showed a trend towards a higher thermal gradient. Cardiopulmonary bypass can also modify systemic vascular resistance, especially in case of deep hypothermic circulatory arrest, but in our study, CPB duration had no influence on thermal gradient (28–30). In a recent study, we observed an inverse gradient in a patient with high dose of milrinone suggesting that the impact of the drug can be quantitatively assessed using temperature gradient by thermography (31).

This study has several strengths. As a single center study there was consistency in protocols for patient management that might not be observed across multiple units. Moreover, all the patients were operated by the same surgeon. We also used a standardized protocol to capture thermal pictures in agreement with medical thermology recommendations and the American Academy of Thermology guidelines. This was crucial to study the correlation between thermal gradient and O_2_ER.

However, there were several limitations. The performance limitations of our thermal camera are the first limitation. The third generation thermal cameras used in the medical field must meet specific criteria (11). The cameras currently used have a thermal resolution of 0.05 °C. Our camera FLIR One Pro may not have been powerful enough, with a thermal sensitivity of 0.07 °C and a rather weak infrared and spatial resolution. We positioned the camera between 80 and 100 cm from the patient to compensate for this, but depending on the patient's age, the area of interest (internal epicanthus) was very small, with a risk of decreased precision of the temperature's measurement. This site has been described as having a good correlation with core temperature, but Teunissen et al. found that it was unreliable in cases of fever or in conditions of physical effort when compared with esophageal temperature (14). However, this was not the case in our study. The second limitation is due to sample size. We had estimated that we needed 120 thermal pictures (3 photos per patient), but several thermal pictures did not meet sufficient quality criteria to be analyzed. A third limitation was the small number of patients with low cardiac output i.e., high O_2_ER. This was probably related to the exclusion of newborns weighing less than 3.5 kg. We excluded them because they were placed in heated incubators that were interfering with thermal recording. Indeed, only a few patients with cardiac malformations typically addressed surgically in the neonatal period, such as conotruncal lesions, or anomalies of the aortic arch were included in our study. However, neonates undergoing surgery for these lesions often have more hemodynamic instability postoperatively and thus higher O_2_ER (32). Another issue related to small sample size was the inability to classify the thermal pictures according to the elapsed time after surgery. Since low cardiac output syndrome generally occurs between 6 h–18 h post-surgery, it would have been interesting to assess the evolution of the correlation between O_2_ER and thermal gradient over time, but there were not enough thermal photos taken in order to do so. Finally, few patients had significant doses of vasoactive drugs, so we could not evaluate their impact on the thermal gradient.

## 5. Conclusion

In this pilot study, we determined thermal gradient in 41 pediatric patients following cardiac surgery. The data collection and image analysis barriers suggest that the image acquisition method needs to be improved before being clinically incorporated into noninvasive multimodal monitoring after cardiac surgery. As technology evolves, we plan to repeat the study using a more powerful camera that will be permanently installed at the patient's bedside. We will then be able to automatically monitor the evolution of the thermal gradient over time and according to changes in the patient's management, reflecting more accurately what is done in clinical practice.

## Data Availability

The raw data supporting the conclusions of this article will be made available by the authors, without undue reservation.
